# Role of Reactive Oxygen Species in the Radiation Response of Human Hematopoietic Stem/Progenitor Cells

**DOI:** 10.1371/journal.pone.0070503

**Published:** 2013-07-25

**Authors:** Masaru Yamaguchi, Ikuo Kashiwakura

**Affiliations:** Department of Radiological Life Sciences, Hirosaki University Graduate School of Health Sciences, Hirosaki, Aomori, Japan; Kagoshima University Graduate School of Medical and Dental Sciences, Japan

## Abstract

Hematopoietic stem/progenitor cells (HSPCs), which are present in small numbers in hematopoietic tissues, can differentiate into all hematopoietic lineages and self-renew to maintain their undifferentiated phenotype. HSPCs are extremely sensitive to oxidative stressors such as anti-cancer agents, radiation, and the extensive accumulation of reactive oxygen species (ROS). The quiescence and stemness of HSPCs are maintained by the regulation of mitochondrial biogenesis, ROS, and energy homeostasis in a special microenvironment called the stem cell niche. The present study evaluated the relationship between the production of intracellular ROS and mitochondrial function during the proliferation and differentiation of X-irradiated CD34^+^ cells prepared from human placental/umbilical cord blood HSPCs. Highly purified CD34^+^ HSPCs exposed to X-rays were cultured in liquid and semi-solid medium supplemented with hematopoietic cytokines. X-irradiated CD34^+^ HSPCs treated with hematopoietic cytokines, which promote their proliferation and differentiation, exhibited dramatically suppressed cell growth and clonogenic potential. The amount of intracellular ROS in X-irradiated CD34^+^ HSPCs was significantly higher than that in non-irradiated cells during the culture period. However, neither the intracellular mitochondrial content nor the mitochondrial superoxide production was elevated in X-irradiated CD34^+^ HSPCs compared with non-irradiated cells. Radiation-induced gamma-H2AX expression was observed immediately following exposure to 4 Gy of X-rays and gradually decreased during the culture period. This study reveals that X-irradiation can increase persistent intracellular ROS in human CD34^+^ HSPCs, which may not result from mitochondrial ROS due to mitochondrial dysfunction, and indicates that substantial DNA double-strand breakage can critically reduce the stem cell function.

## Introduction

Mitochondria, the organelles that produce the energy molecule adenosine triphosphate by oxidative phosphorylation, are also a source of reactive oxygen species (ROS) such as superoxide and hydrogen peroxide, which are generated during respiratory metabolism *in vivo*. Mitochondria contain extranuclear genomes that encode proteins constituting the mitochondrial electron transport chain. This series of proteins produces adenosine triphosphate by electron transfer and generates ROS as a byproduct [1]–[3]. The abnormal accumulation of endogenous or exogenous ROS by the mitochondrial electron transport chain or in response to low linear energy transfer ionizing radiation, such as X-rays, may cause lipid peroxidation, protein denaturation, or DNA mutations [4]–[6].

Hematopoietic stem/progenitor cells (HSPCs), which are present in small numbers in hematopoietic tissues, can self-renew to maintain their own undifferentiated phenotype and can differentiate into all functional mature hematopoietic cells. Hematopoietic stem cells are usually present in a special microenvironment called the stem cell niche, which maintains the stemness by controlling the ROS generation [7]–[10]. In this niche, physiological levels of intracellular ROS affect the endogenous growth signals, cell survival, proliferation, and differentiation of HSPCs with the production of many cytokines [2], [11], [12]. When the ROS are elevated beyond physiological levels, oxidative stress can cause HSPC dysfunction and aging. These cells are extremely sensitive to oxidative stressors, such as anti-cancer agents, radiation, and the extensive accumulation of ROS [13]–[19]. In addition, because hematopoietic stem cells generally reside in the G_0_ phase of the cell cycle and require very little energy, they possess lower intracellular mitochondrial contents than other functional mature cells [20]–[24]. Recently, Charlie et al. reported that CD34^+^ hematopoietic stem cells with a low mitochondrial mass are enriched in hematopoietic repopulating stem cell function, and that the upregulation of nascent mitochondrial biogenesis in CD34^+^ HSPCs parallels the loss of pluripotency [25]. Thus, the response of mitochondrial function and intracellular ROS to ionizing radiation may also depend on the radiosensitivity of CD34^+^ HSPCs. However, information about the relationship between the radiosensitivity of HSPCs and mitochondrial function is limited.

To determine the contributions of mitochondria and intracellular ROS to the radiosensitivity of human HSPCs, the present study investigated the features of X-irradiated CD34^+^ HSPCs prepared from human placental/umbilical cord blood and cultured with hematopoietic cytokines. We herein describe the influence of radiation on the HSPC proliferation and differentiation from the perspective of mitochondrial function.

## Results

### Characteristics and Radiosensitivity of Myeloid Hematopoietic Progenitors

**Figure 1:**
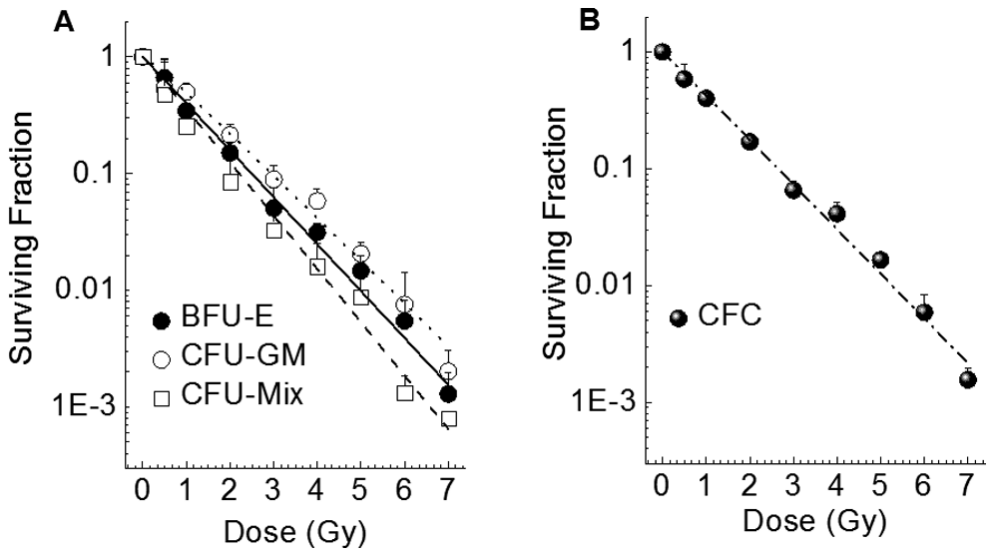
The radiation dose–response curves of human umbilical myeloid hematopoietic progenitors. CD34^+^ HSPCs were X-irradiated and assayed in methylcellulose cultures for 14 days. The values represent the means ± SD of three separate experiments performed in three wells. Curves were fitted as described in the Materials and Methods. Panel A and B: Radiation dose-response curves for each of the cell types and for the total hematopoietic myeloid progenitor cells were shown, respectively.

To evaluate the radiosensitivity of myeloid hematopoietic progenitors, CD34^+^ HSPCs exposed to 0.5–7 Gy were assayed for burst-forming unit-erythroid (BFU-E); colony-forming unit-granulocyte macrophage (CFU-GM); colony-forming unit-granulocyte erythroid, macrophage, and megakaryocyte (CFU-Mix); and colony-forming cells (CFCs) using methylcellulose semisolid culture supplemented with a hematopoietic cytokine combination comprising recombinant human erythropoietin (EPO), granulocyte colony stimulating factor (G-CSF), granulocyte/macrophage colony stimulating factor (GM-CSF), interleukin-3 (IL-3) and stem cell factor (SCF). The radiation survival curves for each type of progenitor cell are shown in Fig. 1. In addition, a single-hit multitarget equation was used to determine the values for *D_0_* and *n*, which are important parameters of these curves that characterize the radiosensitivity, and these are summarized in Table 1. The parameter *D_0_* is the dose that reduces the survival rate up to 37%, and *n* is the number of targets in the cell under the single-hit multi-target theory. In general, the values for *D_0_* and *n* range from 1 to 2 Gy and from 1 to 10, respectively. Progenitor-derived colony formation was found to decrease with dose. The *D_0_* values ranged from 0.95 to 1.19. No shoulders were observed on the dose–response curves, and the *n* values ranged from 1.01 to 1.20, suggesting that CD34^+^ HSPCs are highly radiosensitive.

**Table 1:** The radiosensitivity of the hematopoietic myeloid progenitor cells.

	*D_0_*	*n*
CFU-GM	1.19±0.11	1.20±0.13
BFU-E	1.08±0.08	1.01±0.14
CFU-Mix	0.95±0.08	1.03±0.14
CFC	1.14±0.10	1.03±0.10

*Note.* The values for *D_0_* and *n* are the 37% survival dose and number of targets, respectively.

### Characteristics and Radiosensitivity of HSPCs

**Figure 2:**
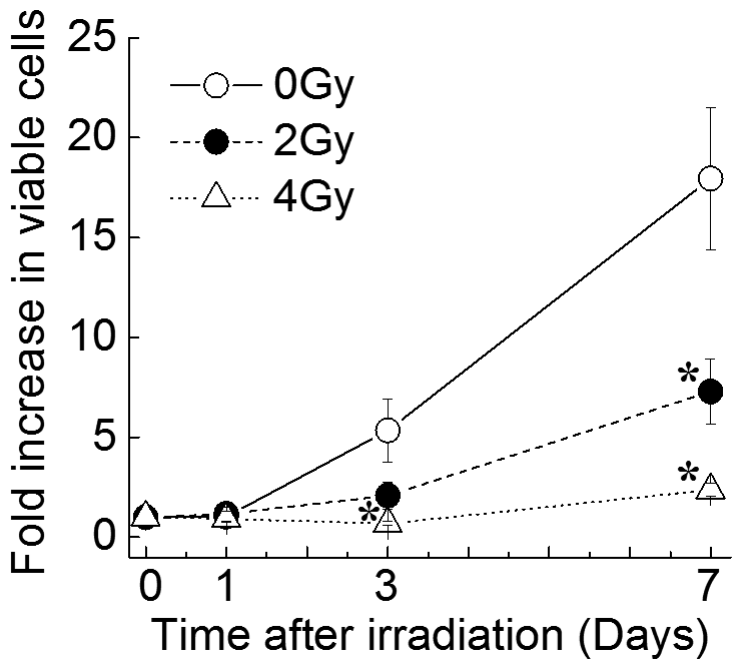
The relationship between the incubation period and ratio of viable cells. CD34^+^ HSPCs exposed to 0, 2 or 4 Gy of X-rays were cultured in liquid medium supplemented with a combination of G-CSF, GM-CSF, IL3, SCF and EPO. Cells were harvested from the culture on days 1, 3 and 7. The viable cells were counted using trypan blue dye exclusion and the results are relative to the initial value. The values represent the means ± SD of three separate experiments. * *P*<0.05 vs. non-irradiated controls.

CD34^+^ HSPCs were cultured in serum-free liquid medium as described in the Materials and Methods to investigate the effects of X-irradiation on the cell proliferation and differentiation. Non-irradiated CD34^+^ HSPCs exhibited an increase in the total number of cells with the duration of culture, proliferating approximately 18-fold from the initial input to day 7 (Fig. 2). Although slight proliferation was observed in the CD34^+^ HSPCs exposed to 2 or 4 Gy of X-rays, the treated cells displayed significant differences in proliferation compared with non-irradiated CD34^+^ HSPCs on days 3 and 7. The total number of myeloid hematopoietic progenitors generated in both cultures was evaluated by calculating the total cell numbers and colony numbers in the cultures at each incubation time. The total number of CFCs generated in the non-irradiated CD34^+^ HSPCs increased in the range from 1.5-fold to 9.3-fold with increasing culture time (Table 2). The CD34^+^ HSPCs exposed to 2 Gy of X-rays exhibited a slight increase on days 3 and 7, showing 1.6- and 2.8-fold increases, respectively. No increase was observed in CD34^+^ HSPCs exposed to 4 Gy.

**Table 2:** The total numbers of myeloid hematopoietic progenitors generated in liquid culture.

Dose (Gy)		Day 0	Day 1	Day 3	Day 7
0 Gy	CFU-GM	778 (1.0)	1,043 (1.3)	4,173 (5.4)	4,565 (5.9)
	BFU-E	913 (1.0)	1,447 (1.6)	9,835 (10)	10,915 (12)
	CFU-Mix	15 (1.0)	72 (4.7)	418 (27)	362 (24)
	CFC	1,706 (1.0)	2,562 (1.5)	14,426 (8.5)	15,842 (9.3)
2 Gy	CFU-GM	–	381 (0.5)	671 (0.9)	892 (1.1)
	BFU-E	–	589 (0.7)	1,920 (2.1)	3,501 (3.8)
	CFU-Mix	–	28 (1.9)	138 (9.1)	404 (26)
	CFC	–	998 (0.6)	2,729 (1.6)	4,797 (2.8)
4 Gy	CFU-GM	–	97 (0.1)	78 (0.1)	81 (0.1)
	BFU-E	–	159 (0.2)	233 (0.3)	253 (0.3)
	CFU-Mix	–	6 (0.4)	13 (0.9)	28 (1.8)
	CFC	–	262 (0.2)	323 (0.2)	362 (0.2)

*Note.* The values in the parentheses indicate the rates of the number of each myeloid hematopoietic progenitor compared to Day 0.

In addition, the effects of radiation exposure on the composition ratios of hematopoietic progenitors generated in liquid culture are summarized in Table 3. With increasing culture time, the BFU-E ratio exhibited an increase similar to that of CFU-GM for each day and dose. In contrast, compared to the initial input, the ratio of CFU-Mix to CFU-GM in non-irradiated and 2 and 4 Gy irradiated cultures exhibited increases of 7.5-, 17- and 6-fold, respectively, on day 7. This suggested that X-irradiated CD34^+^ HSPCs treated with hematopoietic cytokines had dramatically suppressed cell growth and clonogenic potential.

**Table 3:** The composition ratios of myeloid hematopoietic progenitors compared to CFU-GM.

Dose (Gy)		Day 0	Day 1	Day 3	Day 7
0 Gy	BFU-E	1.28 (1.0)	1.57 (1.2)	2.70 (2.1)	2.96 (2.3)
	CFU-Mix	0.02 (1.0)	0.09 (4.5)	0.10 (5.0)	0.15 (7.5)
2 Gy	BFU-E	–	1.77 (1.4)	3.61 (2.8)	3.98 (3.1)
	CFU-Mix	–	0.09 (4.5)	0.20 (10)	0.35 (17)
4 Gy	BFU-E	–	1.75 (1.4)	3.06 (2.4)	2.72 (2.1)
	CFU-Mix	–	0.06 (3.0)	0.17 (8.5)	0.12 (6.0)

*Note.* The values in the parentheses indicate the composition ratios compared to Day 0.

### Detection of Intracellular ROS and Mitochondrial Superoxide Production

**Figure 3:**
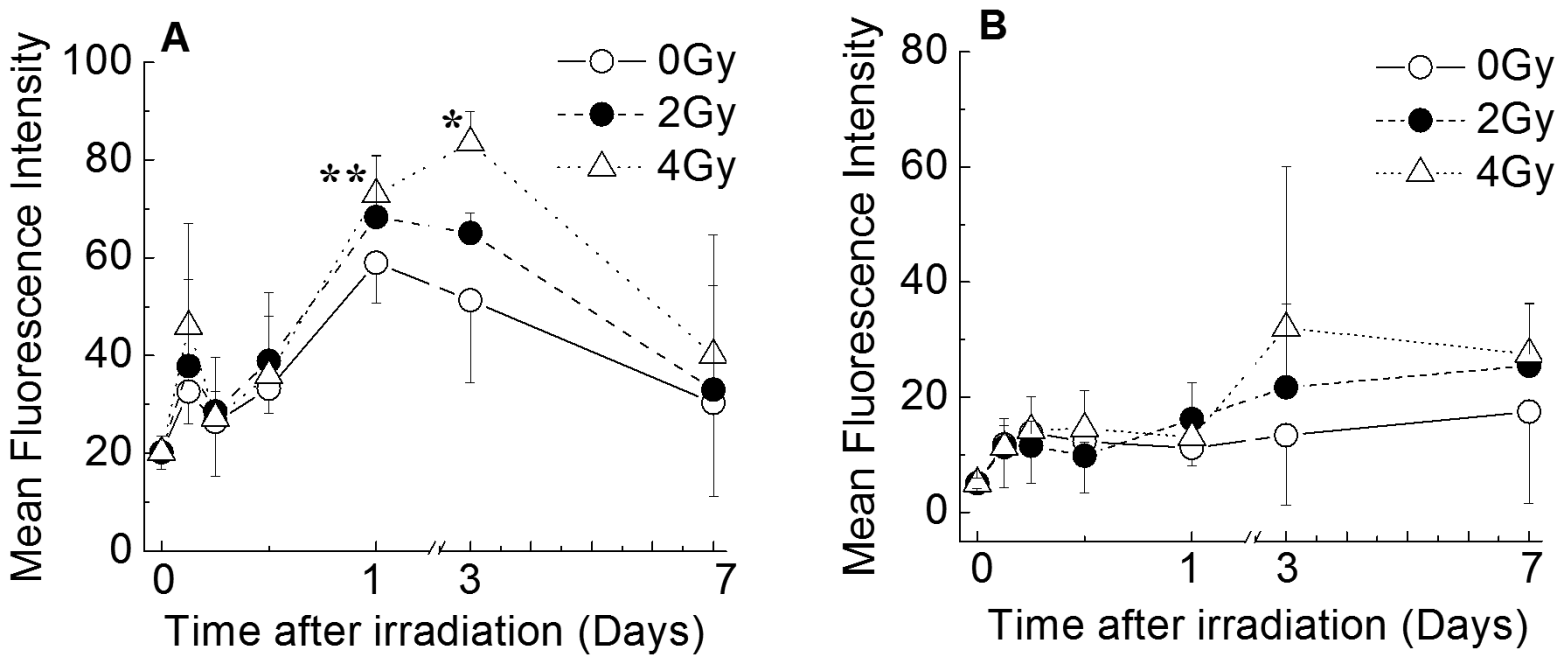
The relationships among the incubation periods, intracellular ROS and mitochondrial superoxide detected in CD34^+^ HSPCs. Cells were cultured for 3 h, 6 h, 12 h, 1 day, 3 days or 7 days. The intracellular ROS (panel A) and mitochondrial superoxide levels (panel B) were analyzed by flow cytometry. The values represent the means ± SD of three separate experiments. * *P*<0.05 or ** *P*<0.01 vs. non-irradiated controls.

The expression levels of intracellular ROS and mitochondrial superoxide were analyzed by flow cytometry to investigate the effects of X-irradiation on the generation of various ROS (Fig. 3). The intracellular ROS generation in non-irradiated CD34^+^ HSPCs reached a maximum level on day 1 and then gradually decreased with increasing culture time (Fig. 3A). CD34^+^ HSPCs exposed to 4 Gy of X-rays displayed a significant increase in intracellular ROS compared with controls on days 1 and 3, with values peaking on day 3 (an approximately 4-fold increase compared to the initial value). Mitochondrial superoxide production did not differ by culture treatment or culture time from days 0 to 7 (Fig. 3B). On day 3, an approximately 7-fold increase compared to the initial input was observed in the culture exposed to 4 Gy. These findings show that the overproduction of intracellular ROS was induced following X-irradiation of CD34^+^ HSPCs, but that this was not derived from the mitochondria.

### Intracellular Mitochondrial Contents

**Figure 4:**
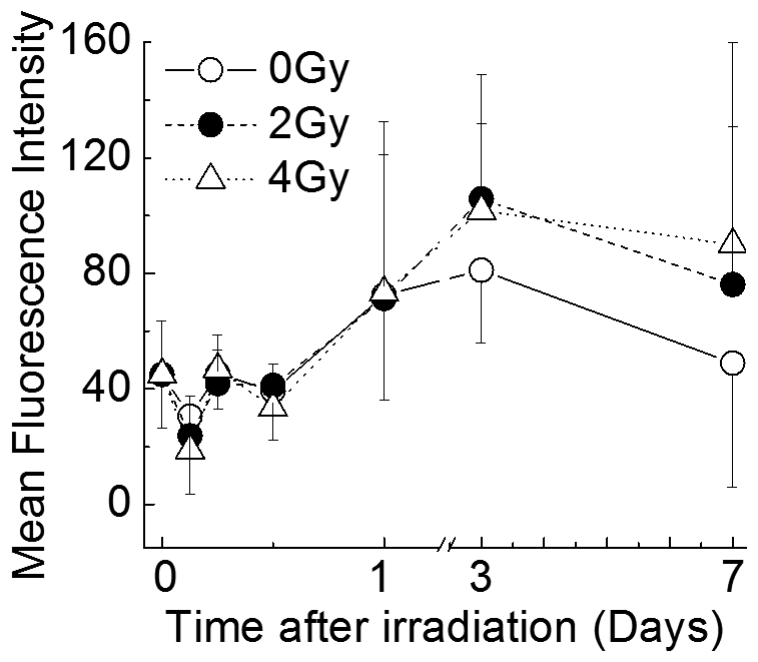
The relationship between the incubation period and intracellular mitochondrial contents detected in CD34^+^ HSPCs. Cells were cultured for 3 h, 6 h, 12 h, 1 day, 3 days or 7 days. After cultivation, the intracellular mitochondrial contents were analyzed by flow cytometry. The values represent the means ± SD of three separate experiments.

The intracellular mitochondrial contents in the cells were measured by flow cytometry to investigate the effects of X-irradiation on nascent mitochondrial biogenesis (Fig. 4). The intracellular mitochondria levels in non-irradiated CD34^+^ HSPCs peaked on day 3 (an approximately 2.5-fold increase compared to the initial input) then decreased slightly thereafter. The X-irradiated cultures exhibited similar levels of mitochondrial contents; no significant differences were observed at two exposure levels, suggesting that X-irradiation does not affect the mitochondrial contents in CD34^+^ HSPCs.

### Gamma-H2AX Expression

**Figure 5:**
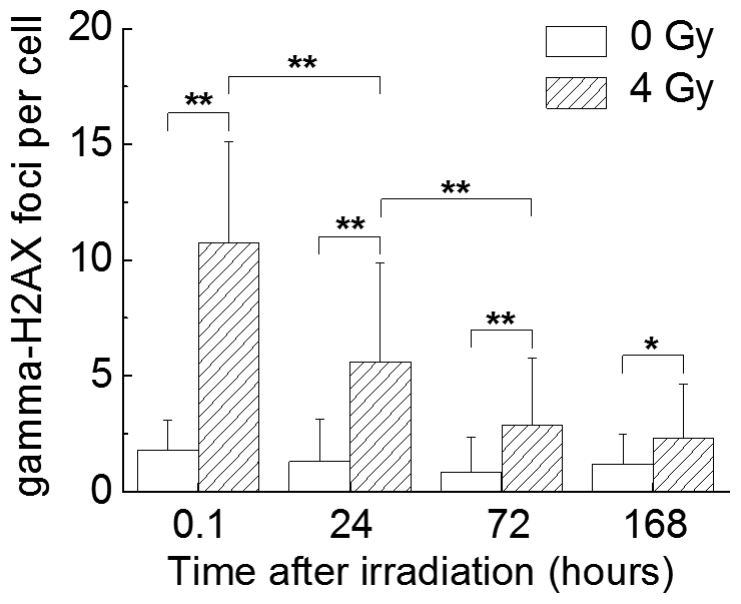
The effects of X-irradiation on DNA double-strand breaks in CD34^+^ HSPCs. CD34^+^ HSPCs exposed to 0 or 4 Gy of X-rays were cultured in liquid medium supplemented with a combination of G-CSF, GM-CSF, IL3, SCF and EPO. Cells were harvested at 24, 78 and 168 hours, and the expression of gamma-H2AX was evaluated as described in the Materials and Methods. * *P*<0.05 or ** *P*<0.01 vs. non-irradiated controls and vs. X-ray irradiated cells.

To monitor the repair of DNA double-strand breaks induced by ionizing radiation, the expression of gamma-H2AX, a marker of the DNA double-strand break response [26], [27], was measured in cells harvested from liquid culture. Radiation-induced gamma-H2AX expression was observed immediately after exposure to 4 Gy of X-rays and gradually decreased over 78 hours (Fig. 5), suggesting that the DNA double-strand breaks induced immediately after irradiation were gradually repaired during the culture period, but that DNA damage remained.

## Discussion

The present study investigated the involvement of intracellular ROS and mitochondria in the proliferation and differentiation of X-irradiated human CD34^+^ HSPCs. The survival curve of progenitor cells demonstrated that HSPCs are sensitive to ionizing radiation (Fig. 1). Similarly, the generation of hematopoietic progenitor CFCs from primitive HSPCs decreased with the radiation dose in liquid culture (Table 2) suggesting that CD34^+^ HSPCs are highly radiosensitive. However, no dose dependence of the composition ratios of hematopoietic progenitors was observed.

The hematopoietic system is generally regulated by several factors in the niche microenvironment, including hematopoietic cytokines and the physiological level of ROS. In the present study, the intracellular ROS production peaked in non-irradiated CD34^+^ HSPCs on day 1 (Fig. 3A). Given that hematopoietic cytokines induce intracellular ROS production, the findings of the present study are consistent with those of previous studies [2], [11]. The physiological level of ROS can lead to activation of the Janus kinase 2, signal transducers activator of transcription 5, Ras/mitogen-activated protein kinase kinase/extracellular signal-regulated kinase, and phosphoinositide 3-kinase/Akt signaling pathways and to the transactivation of cytokine receptors and cell cycle progression, and can promote cell proliferation and differentiation. [11], [28]–[31]. However, the production of excessive ROS triggered by ionizing radiation or chemotherapeutic agents can indirectly damage cellular components and DNA [13]–[16], [32].

In the present study, CD34^+^ HSPCs exposed to 4 Gy of X-rays showed a significant increase in ROS production on days 1 and 3 in liquid culture (Fig. 3A). However, the cultures irradiated with 2 Gy were similar to non-irradiated CD34^+^ HSPCs with respect to ROS generation (Fig. 3A). In addition, the mitochondrial superoxide levels were similar for all cultures (Fig. 3B). The mitochondrial contents gradually increased in all cultures until day 3, and no significant differences were observed (Fig. 4). However, the increases of the mitochondrial contents were not related to the cell growth (Fig. 1). In addition, although a statistically significant increase of gamma-H2AX expression was observed in the irradiated cells, it gradually decreased with the time after irradiation (Fig. 5). We did not check whether DNA repair occurred promptly after irradiation or whether gamma-H2AX expression was observed only in the living cells. However, it is generally thought that DNA double-strand breaks that are induced immediately after irradiation are gradually repaired during the culture period [33], while DNA damage remains. In any case, since the contents of hematopoietic progenitors in the cells generated in the liquid culture were decreased in a radiation dose-dependent manner (Table 2), our findings suggest that the DNA damage caused by ionizing irradiation leads to a critical loss of clonogenic potential. Further studies will be required to determine the precise mechanisms underlying these issues. The findings regarding the mitochondrial contents were consistent with the production of mitochondrial superoxide, and it is considered that the intracellular ROS induced by X-irradiation may not be derived from mitochondria.

The DNA double-strand break response in cultures was assessed by measuring the gamma-H2AX expression in CD34^+^ HSPCs exposed to 4 Gy of X-rays in liquid culture (Fig. 5). It was not possible to detect gamma-H2AX expression in non-irradiated CD34^+^ HSPCs, but the expression increased to detectable levels immediately after exposure to 4 Gy of X-rays and then decreased gradually over 78 hours (Fig. 5). Yahata et al. reported that the induced elevation of ROS within CD34^+^ HSPCs, as determined using a glutathione synthesis inhibitor (buthionine sulfoximine), was associated with extensive DNA damage [14]. Elevated DNA damage activates the expression of cell cycle inhibitors in HSPCs, leading to premature senescence and the eventual loss of stem cell function [14], [34]. Wang et al. reported that total body irradiation in mice induces selective and persistent oxidative stress in hematopoietic stem cells, partly via the upregulation of the membrane-bound NADPH oxidase 4 [35]. In addition, Yamamori et al. reported that ionizing radiation induces mitochondrial ROS production in human lung carcinoma A549 cells, which is accompanied by elevated mitochondrial contents and upregulation of the mitochondrial electron transport chain function [36]. However, we observed no increase in the mitochondrial contents or ROS with the increase in cell numbers generated in liquid culture (Figs. 2, 5), suggesting that the DNA damage induced by the X-irradiation of CD34^+^ HSPCs accounts for much of the cellular damage observed in the presence of hematopoietic cytokines. Moreover, uncontrolled overproduction of intracellular ROS in CD34^+^ HSPCs leads to abnormal hematopoiesis and HSPC dysfunction [14], [34], suggesting that an increase in the physiological level of ROS by X-irradiation is involved in the suppression of HSPC proliferation and differentiation (Fig. 2, Table 2). We accordingly propose that the NADPH oxidase family is primarily responsible for the X-irradiation-induced persistent and prolonged intracellular ROS generation in human CD34^+^ HSPCs, and that the resulting oxidative stress is associated with inhibition of the clonogenic potential of HSPCs. Our results indicate that damaging effects induced by mitochondrial ROS are limited.

The present study has demonstrated that X-irradiation-mediated damage to CD34^+^ HSPCs, as measured by decreases in the clonogenic and proliferative potential, is induced to some extent by intracellular ROS generation. Substantial DNA damage may lead directly to a loss of HSPC function. Although physiological levels of intracellular ROS play an important role in endogenous growth signaling, cell survival, and the proliferation and differentiation of HSPCs, complete elucidation of the role of ROS in hematopoiesis awaits additional investigation of the DNA repair mechanisms that are active in HSPCs.

## Materials and Methods

### Growth Factors and Fluorescent Antibodies

Recombinant human IL-3 and human SCF were purchased from BioSource (Tokyo, Japan). Recombinant human EPO and G-CSF were purchased from Sankyo Co. Ltd. (Tokyo, Japan). Recombinant human GM-CSF was purchased from PeproTech, Inc. (Rocky Hill, NJ, USA). These growth factors were administered at the following concentrations: IL-3, 100 ng/ml; SCF, 100 ng/ml; EPO, 4 U/ml; G-CSF, 10 ng/ml and GM-CSF, 10 ng/ml medium. The following fluorescence-labeled monoclonal antibodies (mAbs) were purchased from Beckman Coulter Immunotech (Marseille, France): fluorescein isothiocyanate (FITC)-conjugated anti-human CD34 (CD34-FITC), phycoerythrin (PE)-conjugated anti-human CD34 (CD34-PE) and mouse IgG_1_-FITC and IgG_1_-PE, which were used as isotype controls. The ROS detection reagent, 5-(and-6)-chloromethyl-2′,7′-dichlorodihydrofluorescein diacetate, acetyl ester (CM-H_2_DCFDA); the MitoSOX™ Red mitochondrial superoxide indicator and the MitoTracker Green FM mitochondrion-selective probe reagent (specially packaged) were purchased from Molecular Probes (CA, USA). Anti-phospho-histone H2AX monoclonal antibodies (JBW301) were purchased from Upstate Biotechnology (NY, USA), and Alexa Fluor 488®-conjugated anti-mouse IgG secondary antibodies were purchased from Molecular Probes.

### Collection, Purification and Cryopreservation of CD34^+^ HSPCs

This study was approved by the Committee of Medical Ethics of Hirosaki University Graduate School of Medicine (Hirosaki, Japan). Informed consent was obtained from mothers who had delivered full-term infants after written and verbal explanations were provided before delivery. Signatures were obtained from the mothers, as well as from a relative, such as the husband or the patient’s mother/father etc. All documents related to the informed consent process were reviewed and approved. After delivery, placental/umbilical cord blood samples were collected into sterile collection bags (CBC-20; Nipro, Osaka, Japan) containing the anticoagulant, citrate phosphate dextrose, according to the guidelines of the Tokyo Cord Blood Bank (Tokyo, Japan). The samples were isolated and maintained separately prior to each experiment. Within 24 hours of cord blood collection, light-density mononuclear cells were separated by centrifugation in a Limphosepar I (1.077 g/ml; Immuno-Biological Laboratories, Takasaki, Japan) instrument for 30 min at 300 *g* and were washed three times with calcium- and magnesium-free phosphate-buffered saline (PBS (−); Sigma-Aldrich, Stockholm, Sweden) containing 5 mM ethylenediamine-N,N,N′,N′-tetraacetic acid (Wako, Tokyo, Japan). The cells were then processed for CD34^+^ enrichment according to the manufacturer’s instructions. An autoMACS™ Pro Separator (Miltenyi Biotec GmbH, Bergisch Gladbach, Germany) was used for the sorting of CD34^+^ cells. The CD34-enriched cell population is referred to as HSPCs in this study. The CD34^+^ HSPCs were suspended in serum-free Cell Banker 3 reagent (Juji Field, Tokyo) and were frozen at −150°C prior to experimentation.

### *In vitro* Irradiation

CD34^+^ HSPCs were exposed to X-rays in serum-free medium within 24 hours of isolation. Radiation (2 or 4 Gy, 150 kVp, 20 mA; 0.5 mm aluminum and 0.3 mm copper filters) was delivered using an X-ray generator (MBR-1520R; Hitachi Medical Co., Tokyo, Japan) with a distance of 45 cm between the focus and the target. The dosage was monitored using a thimble ionization chamber placed next to the sample during irradiation. The dose rate was approximately 1 Gy/min.

### Methylcellulose Culture

The lineage-committed myeloid hematopoietic progenitor cells included CFU-GM, BFU-E and CFU-Mix cells. CD34^+^ HSPCs were assayed in methylcellulose culture as described previously [37] by suspending them in 1 ml of methylcellulose medium (MethoCult H4230, StemCell Technologies Inc.) supplemented with a hematopoietic cytokine mixture that contained five growth factors (GFs; IL-3, SCF, EPO, G-SCF, and GM-SCF). This mixture was transferred to 24 well cell culture plates (Falcon, Becton Dickinson Biosciences, Franklin Lakes, NJ) at 0.3 ml/well and then was incubated for 14 days at 37°C in a humidified atmosphere with 5% CO_2_. Colonies consisting of more than 50 cells were counted using an inversion microscope (Olympus, Tokyo, Japan).

### Liquid Culture

Frozen cells were thawed rapidly at 37°C, suspended in Cell Lotion (Juji Field, Tokyo, Japan) and centrifuged at 400 *g* at 10^○^C for 10 min. The supernatant was removed and resuspended in serum-free Iscove’s Modified Dulbecco’s Medium (Gibco Invitrogen, Grand Island, NY, USA) supplemented with BIT 9500 serum substitute (StemCell Technologies, Vancouver, Canada) and low density lipoprotein (Calbiochem®, US and Canada). CD34^+^ HSPCs were treated with the hematopoietic cytokine combination of five GFs in γ-ray–sterilized assist tubes (Assist, Tokyo, Japan) at a concentration of 3×10^4^ cells/0.5 ml/tube, and were incubated at 37°C in a humidified atmosphere with 5% CO_2_. The number of viable cells was counted on days 0, 1, 3 and 7 using the trypan blue dye exclusion method (Sigma-Aldrich Co. Ltd., St. Louis, MO, USA).

### Flow Cytometric Analysis

The expression of specific cell surface antigens was analyzed by direct immunofluorescence flow cytometry (FC500, Beckman Coulter Inc., Fullerton, CA, USA) using triple staining combinations of mAbs. In brief, cells were incubated with saturated concentrations of the relevant mAbs for 20 min at room temperature, washed and then subjected to flow cytometry. For each experiment, the isotype-matched control mAb was used as a negative control.

### Measurement of the Intracellular ROS Generation

The fluorescent probe, CM-H_2_DCFDA, and the mitochondrial superoxide indicator, MitoSOX Red, were used for the assessment of intracellular ROS and for mitochondrial superoxide generation, respectively. CD34^+^ HSPCs were incubated for 15 min with 5 µM CM-H_2_DCFDA in PBS (−) and for 10 min with 2.5 µM MitoSOX Red in Hanks’ balanced salt solution (HBSS) (+) at 37°C in a humidified atmosphere with 5% CO_2_. Unincorporated CM-H_2_DCFDA or MitoSOX Red was removed by washing with PBS (−) or HBSS (+). Each sample was resuspended in PBS (−) or HBSS (+) and was analyzed by flow cytometry as described above.

### Measurement of the Intracellular Mitochondrial Fluorescence

The intercellular mitochondrial contents were measured by staining cells with a membrane potential-independent mitochondrial dye; MitoTracker Green FM [38]–[40]. CD34^+^ HSPCs were incubated with 5 nM Mitotracker Green FM in PBS (−) for 15 min at 37°C in a humidified atmosphere containing 5% CO_2_. Unincorporated MitoTracker Green FM was removed by washing the cells with PBS (−). Each sample was resuspended in PBS (−) and then analyzed by flow cytometry as described above.

### Immunofluorescent Detection of Gamma-H2AX

Following treatment of the cells with hematopoietic cytokines and X-irradiation for the indicated periods, CD34^+^ HSPCs (2×10^5^ cells/sample) were harvested, washed with PBS (−) and fixed with ice-cold 75% ethanol for 10 min at room temperature. Fixed cells were washed with PBS (−), permeabilized in 0.5% Triton X-100 (Wako, Osaka, Japan) on ice for 5 min, and washed twice with PBS (−). The cells were then incubated with an anti-phospho-histone H2AX monoclonal antibody diluted 1∶300 with 20 mM Tris-HCl [pH 7.4], 137 mM NaCl, 0.1% Tween-20 (TBST) containing 5% skim milk at 37°C for 120 min, and were subsequently washed with PBS (−) and incubated with Alexa Fluor 488-conjugated anti-mouse IgG secondary antibody diluted 1∶400-fold with TBST containing 5% skim milk at 37°C for 60 min. Following a second wash with PBS (−), the cells were adhered to microscope glass slides (Matsunami Glass Ind., Osaka, Japan) using a StatSpin® CytoFuge 2 (Iris Sample Processing, Inc., MA, USA), and mounted with Vectashield® Mounting Medium with DAPI (Vector Laboratories, Inc., CA, USA). For the quantitative analysis, the gamma-H2AX foci were counted per cell using a LSM 710 laser scanning microscope (Carl Zeiss Microscopy Co., Ltd., Tokyo, Japan) with a Z-stack function that scans by changing the depth for thick samples and storing the data as layers. Under blinded conditions, the gamma-H2AX foci per cell were counted for at least 50 cells in every sample.

### Statistical Analysis

The statistical analysis was performed using the Origin software package (OriginLab® Pro v8.1; Northampton, MA, USA) for the Windows operating system. Dose–survival curves were fitted using the Levenberg–Marquardt algorithm, and the values for *D_0_* (37% survival dose) and *n* (number of targets) were determined using a single-hit multi-target equation. Data were obtained from three independent experiments and were compared between control and experimental groups by the paired t-test. The data were analyzed by two-sided Student’s t-tests and the Mann–Whitney U-test. *P<0.05 and **P<0.01 were considered to be statistically significant.
